# Type, density, and healthiness of food-outlets in a university foodscape: a geographical mapping and characterisation of food resources in a Ghanaian university campus

**DOI:** 10.1186/s12889-022-14266-7

**Published:** 2022-10-13

**Authors:** Daniel O. Mensah, Godwin Yeboah, Michael Batame, Rob Lillywhite, Oyinlola Oyebode

**Affiliations:** 1 Warwick Medical School, Warwick Centre for Global Health, Division of Health Sciences, University of Warwick, Coventry, UK; 2 Technology for Research, Information and Digital Group, University of Warwick, Coventry, UK; 3 Department of Geography and Resource Development, School of Social Sciences, University of Ghana, Accra, Ghana; 4 School of Life Sciences, Warwick Medical School, University of Warwick, Coventry, UK

**Keywords:** University foodscape, Food outlet healthiness, Non-communicable disease, Ghana, Humanitarian OpenStreetMap, Volunteered and collaborative mapping

## Abstract

**Introduction:**

Food environments are viewed as the interface where individuals interact with the wider food system to procure and/or consume food. Institutional food environment characteristics have been associated with health outcomes including obesity and nutrition-related non-communicable diseases (NR-NCDs) in studies from high-income countries. The objectives of this study were (1) to map and characterise the food-outlets within a Ghanaian university campus; and (2) to assess the healthiness of the food outlets.

**Methods:**

Data collection was undertaken based on geospatial open-source technologies and the collaborative mapping platform OpenStreetMap using a systematic approach involving three phases: remote mapping, ground-truthing, and food-outlet survey. Spatial analyses were performed using Quantum Geographical Information System (QGIS) and comprised kernel density, buffer, and average nearest neighbour analyses to assess outlet distribution, density, and proximity. A classification system was developed to assess the healthiness of food-outlets within the University foodscape.

**Results:**

Food-outlets were unevenly distributed over the University foodscape, with many outlets clustered closer to student residencies. Informal food-outlets were the most frequent food-outlet type. Compared to NCD-healthy food-outlets, NCD-unhealthy food-outlets dominated the foodscape (50.7% vs 39.9%) with 9.4% being NCD-intermediate, suggesting a less-healthy university foodscape. More NCD-unhealthy food outlets than NCD-healthy food outlets clustered around student residences. This difference was statistically significant for food outlets within a 100-m buffer (*p* < 0.001) of student residence and those within 100 and 500 m from departmental buildings/lecture halls (at 5% level of significance).

**Conclusion:**

Further action, including research to ascertain how the features of the University’s food environment have or are influencing students’ dietary behaviours are needed to inform interventions aimed at creating healthier foodscapes in the study University and other campuses and to lead the way towards the creation of healthy food environments at the home, work, and community levels.

**Supplementary Information:**

The online version contains supplementary material available at 10.1186/s12889-022-14266-7.

## Introduction

Globally, obesity prevalence tripled between 1975 and 2016 [1] and unhealthy diet supplied by an increasingly unhealthy food environment has been cited as a key culprit. In that, the global food environment, the interface where individuals interact with the wider food system to procure and/or consume food, has in recent decades seen rapid transformations that make unhealthier food options increasingly available. Recent conceptualisations distinguish this as the external food environment from personal food environment domains [2]. The external domain comprises exogenous features such as availability, prices, vendor and product characteristics, marketing, and governance. Conversely, the personal domain is defined to include individual-level factors including physical accessibility, affordability, convenience and desirability [2]. Given that food choices are made within the limits of options the food system makes available, food environments exert significant influences on food-related behaviours [3]. It has for example been suggested that the rapid increase in global obesity prevalence is a materialised reflection of individuals’ natural response to their environment that promotes excess calorie intake and sedentary behaviour [4, 5].

Emerging adults, 18 to 25-year olds, have been found to engage in nutritionally poor and less healthy food behaviours, and especially so in comparison with other age cohorts [6–8]. This is generally true across 28 countries in the European Union [8], Australia [6] and USA [7]. They are less likely to meet standard dietary recommendations [9–12]. Emerging adulthood is a key transition period when individuals establish independence and responsibility for life choices, including autonomy in food- and health-related choice [13, 14]. Emerging adulthood presents an opportune period to influence the adoption of healthy lifestyles, including dietary and physical activity behaviours for immediate and future health and environmental benefits.

The university or school food environment has gained popularity as an important factor shaping young people’s eating habits in studies emanating from high-income countries (HICs) [15–17]. The university is one of the few places a large population of emerging adults live and/or work and spend most (≈35 h/week for ≈4 years) of their emerging adult life. The university food environment therefore offers an ideal setting to positively influence emerging adults’ food behaviours. Many university students live away from home for the first time, where taking charge of their individual food needs becomes a new and often a difficult challenge [18]. The university campus is where certain health behaviours (including food-related) that may perpetuate into adulthood or trigger the onset of obesity and/or other NCDs are nurtured. For example, university food environments have been reported to offer less healthy food options than healthy food options in studies from USA [19, 20] Germany [21], Australia [22], New Zealand [23], Brazil [24], and South Africa [25]. In longitudinal studies, more ‘freshmen’ (i.e.: first years) living on campus were obese or gained significantly more weight at the end of their first year than students with other living arrangements, with 3.38 kg mean weight gain for the subgroup of weight gainers [26, 27]. 

Nearly 10% of the population in sub-Saharan Africa (SSA) go through tertiary education institutions according to [28] based on UNESCO Institute of Statistics 2020 data. Moreover, the emerging adult age-group is particularly important for SSA, which is home to the youngest population, the size of which is projected to double by 2050 [29]. What is more, in SSA and other low-and-middle-income countries (LMICs), there is a rapidly increasing double-burden of obesity and malnourishment linked to unhealthy diets [2, 30, 31]. However, recent systematic reviews of food environment research have found extremely limited evidence of research capturing the features of the prevailing food environment in the sub-region and how this relates to the ongoing changes in food-related behaviours and health outcomes [32, 33]. This is a significant research gap given the fundamental differences between HIC and SSA cultures regarding food value-chains, production, supply environments, food acquisition and consumption practices, and public health nutrition challenges. This study sought to address this lacuna by mapping and characterising the features that constitute the food environment in an urban Ghanaian university campus.

## Objectives

1. To identify and map the distribution of food-outlets within the University of Ghana campus.
2. To ascertain the type of food-outlets that make up the University of Ghana food environment.
3. To assess the healthiness of the various food-outlets distributed over the University of Ghana campus.

## Methods

### Study area

The University of Ghana is the oldest and the largest public university in Ghana, located about 13 km north-east of Accra and with a land size of about 99.3 hectares, including a 23-hectare botanical garden. The University has three campuses, namely Legon (main campus), Korle-Bu and Accra City, which are suburban areas, comprising a total student population size of 39,249 including both undergraduate (85.4%) and graduate (14.6%) students. The majority (97.9%) of the population is Ghanaian, 1.5% of other African nationality and 0.6% of other nationality. The University’s Legon campus has 14 halls of residence (six traditional halls^1^ and eight new residencies^2^ commissioned in 2011), the International Student Hostel I and II, and the Valco Trust Hostel which altogether house about 52% of students of the Legon main and Korle-Bu campuses. The remainder lived in private hostels, rented accommodation from private landlords, and other living arrangements. This study [part of a wider project (34, 35]) covered outlets in and around departmental buildings, on-campus accommodation facilities and accommodation facilities like the African Union, Bani, James Topp Nelson Yankah, and Evandy halls, which are usually classified as off-campus facilities among students due to being distant from central campus. The University operates a collegiate system which includes four colleges namely: the College of Basic and Applied Sciences, College of Education, College of Health Sciences and the College of Humanities. Students spend an average number of six (6) hours/day at their departments/lecture halls.

### Data collection

The data collection employed a systematic approach involving three phases namely (1) remote mapping (2), ground-truthing, and (3) food-outlet survey. The remote mapping phase included online mapping and online validation. There was an initial update of OpenStreetMap (OSM) based on freely available satellite imagery of the study area to create a vector basemap made up of footprints of building structures and routes. All building structures and routes within the boundary of the University campus were remotely mapped and validated online using the Humanitarian OSM Team’s (HOT) Tasking Manager (Tasking Manager is a web-based interface to coordinate mapping task and edit OSM using map editors such as iD editor) to create a basemap which guided ground-truthing or block-by-block observation [36–38].

The ground-truthing activity involves field verification of building structures and routes mapped during the online remote mapping and validation. All data were obtained through ground-truthing survey and direct observation by the first author, and two research assistants recruited for this study, in collaboration with up to 20 well-trained University of Ghana (UG) YouthMapper volunteers stationed at six different clusters of the University campus (namely: the Main campus area; Vice Chancellor’s residence; Athletic oval; Diaspora area; and Botanical gardens; and Pentagon area). Atlases of the verified basemap were generated and printed using a web-based interface (Fieldpapers.org) for generating A4 field paper maps (hereafter, FieldPaper sheets). FieldPaper sheets (FPS) were used to guide the ground-truthing survey and to directly observe and verify the location and typology of all structures in the study area, with particular interest in residential structures and food outlets. Data were recorded using a questionnaire instrument developed using open source software namely OpenDataKit (ODK) [39] and OpenMapKit (OMK) [40] which were loaded into Samsung Galaxy S5 and Alcatel 3 V android mobile phones. The verified structures were also annotated on the FPS for accuracy and as back-up reference when updating OSM. Each FPS has a quick-reference (QR) code which allows scanned FPS to be oriented when overlaid on OSM. The ODK questionnaire was piloted and modified prior to its usage. Volunteers were paired to conduct the actual ground-truthing survey between 8th October and 7th November 2019 by walking through the streets of the entire study area. This systematic approach offered three main benefits: (1) the potential to save time, and (2) comprehensive geographical coverage, (3) mitigation for other inherent weaknesses of individual methods [17, 38]. The study focused on university-managed student residences. Rented rooms from private landlords were excluded, as these were farther from the University campus.

Volunteers were trained to follow a standard protocol as follows. First, the shape on the FPS representing the building structure was traced out in pencil. Where there was a new structure, a representative shape was drawn on the FPS. Structures were assigned serial numbers starting with 1 (as a three-digit number—001) for the first structure visited using a naming convention (detailed in Table 1) that assigned a unique 13-digit structure ID number to each structure. Secondly, the name, location/street address, structure type/use (e.g., residential, classroom, office, or food outlet, etc.), corresponding details on individual structures captured on the FPS were recorded in ODK collect where the unique 13-digit structure identification (ID) is generated. The Geographical Positioning System (GPS) coordinates of the structure were thirdly recorded using the OMK feature in the ODK questionnaire. Finally, a front-view photograph of the structure was taken. Data collected were quality checked by team leaders before submitting to the ODK Server hosted by the Institute for Global Sustainable Development (IGSD) based at the University of Warwick. Identified discrepancies were discussed with volunteers in WhatsApp group chat and at weekly meetings and subsequently corrected.

**Table 1:** Naming convention for assigning unique 13-digit structure ID’s

The naming convention is as follows:
First letter/alphabet unique to the study (‘**N**’ in this case) followed by
Field paper sheet code which should be three characters (this was **A01** in this study) followed by
Three-digit enumeration area code (for e.g., **111** for Pentagon area) followed by
Field worker identification code (3-digits, e.g., **550**) followed by
Last three digits indicated serial numbering of structures, with 1 (entered as **001**) being the first structure visited by the fieldworker
Finally, the unique 13-digit structure ID for the first structure visited, for example, would be **NA01111550001**

Adapted from NIHR Global Health Research Unit, 2018

In the food-outlet survey, a survey instrument was developed based on insights from the Nutrition Environment Measures Survey (NEMS) instruments for restaurants [41] and stores [42] by Glanz and colleagues to assess food outlets (mapped in step 3) within the University of Ghana food environment using ODK collect. The assessment tool captured information on the type of food outlet and food options available—including fruit, vegetables, carbonated/sugar-sweetened beverages and salted snacks, fast-food, and other prepared/cooked foods—opening hours, advertising material, availability of seating, etc. The location of food outlets as captured in step 3 was also validated to ensure accuracy. The assessment tool was pilot-tested in the Main campus and Diaspora clusters with four different volunteer groups and subsequently revised based on a comparison of results, including the classification of food outlets, to ensure accuracy.

A food outlet classification system was developed given the non-existence of a standard food outlet classification regime for the study country. This was based on the literature [5, 23, 43, 44] and the characteristics of the food outlets. Food outlets were initially classified into two broad categories—food stores and food service places—based on the service type. These were further divided based on the features of the structure or edifice the food outlet operated from (i.e., movable/permanent, size, seating availability and type, number of vendors, etc.), key aspects of business practice (i.e., self-service, take-away/delivery service, operating hours, etc.), and type/variety of foods. Appendix 1 shows the typology of food outlets in the University foodscape and the description of each outlet type.

As the NEMS concept (employed in the development of the food-outlet survey instrument) enables the capturing of both healthy and unhealthy food options available at eating venues, food outlets were also categorised as either NCD-healthy, NCD-intermediate, or NCD-unhealthy based on:

1. the level of processing of the food options on offer [45];
2. whether or not the food options on offer are known risk factors for obesity, hypertension, other cardiovascular or NCDs [46–48]; and
3. whether or not the food options are known to offer protection against NCDs (after Maimaiti et al., 2020) [46–48].

The NCD-unhealthy food outlets category encompassed those that sold no fruit and/or vegetable choices, they offered only ultra-processed foods (UPFs), high-fat, and energy-dense choices that encourage excess calorie intake. NCD-healthy food outlets included outlets that had the highest proportion of food options being FV, other plant-based food options, and low-fat food choices or food option that have been associated with healthy eating [47, 48]. The NCD-intermediate category comprised of food outlets that did not fit neatly into either of the NCD-healthy or NCD-unhealthy categories and the contribution of the foods/food outlet types to BMI, hypertension, or other NCDs is inconclusive. See Table 2 for the summary of the criteria.

**Table 2:** Food outlet healthiness classification and definition as NCD-healthy, NCD-unhealthy, or NCD-intermediate

Outlet healthiness category	Definition
NCD-healthy	This included outlets that had FV, other plant-based food options, organic foods, and low-fat food choices on offer or have been associated with healthy eating [47, 48]. E.g., Store/stall/table-top vendor specialising in selling fresh fruit and/or vegetable options only; Organic food store/stall/table-top vendor specialising in stocking only fresh organic fruit, vegetable, and other plant-based food options; Store/shop/stall/table-top vendor selling drinking water only; Fruit juice/smoothie/puree stand; Food service places (Restaurants) serving vegetable soups/sauces/stews, legume soups/stews/sauces, and vegetable salads as main part of menu
NCD-unhealthy	This encompassed food outlets that sold no fruit and/or vegetable choices, they offered ultra-processed foods (UPFs), high-fat, and energy-dense choices that encourage excess calorie intake (Costa et al., 2019 [49]; Costa et al., 2018 [50]; Nardocci et al., 2019 [51]; Piernas et al., 2016 [52]; Rauber et al., 2018 [53], 2020 [54]; WCRF/AICR, 2018 [55]). E.g., Store/stall/table-top vendors selling confectionery, carbonated/SSBs or drinks; ice creams; sugared/salted snacks including cookies, cakes, and biscuits; frozen pizza; jams; bouillon/stock cubes or powders; packaged instant noodles, salted fish/meat [55], blended kenkey (ice-kenkey). Food service places offering stir-fried rice, instant noodles, *kelewele*, deep-fried foods [56], sausages, khebab/other processed meat/salted meat, salted fish, burgers, hotdogs, chicken nuggets [55, 57], alcoholic drinks, milk shake
NCD-intermediate	This included food outlets that did not fit neatly into either of the NCD-healthy or NCD-unhealthy categories and the contribution of the foods/food outlet types to obesity/overweight, hypertension, or other NCDs is inconclusive or stocked proportionate mixture of foods known to be NCD-healthy as well as food known to be NCD-unhealthy

### Data analysis

Geocoding and a food retail environment spatial distribution analysis were undertaken using Quantum Geographical Information System (QGIS) Desktop software version 3.10.0 with Geographic Resources Analysis Support System (GRASS) software version 7.6.1 and Microsoft Excel Spreadsheet. The density of the various outlets per kilometer square over the University foodscape was assessed. In a nearest-neighbor analysis, the distance between two outlets of the same type was determined. Distance to nearest hub (points) analysis was applied to determine the distance between food outlets and classrooms, and between outlets and students’ residences. Two-sample t-tests were ran in MS Excel Spreadsheet to compare the differences between NCD-healthy and NCD-unhealthy food outlets in terms of their proximity to student residencies. The same statistical analysis was ran to compare the proximity of NCD-health outlets and that of NCD-unhealthy food outlets to departmental buildings/lecture halls.

## Results

**Fig. 1:**
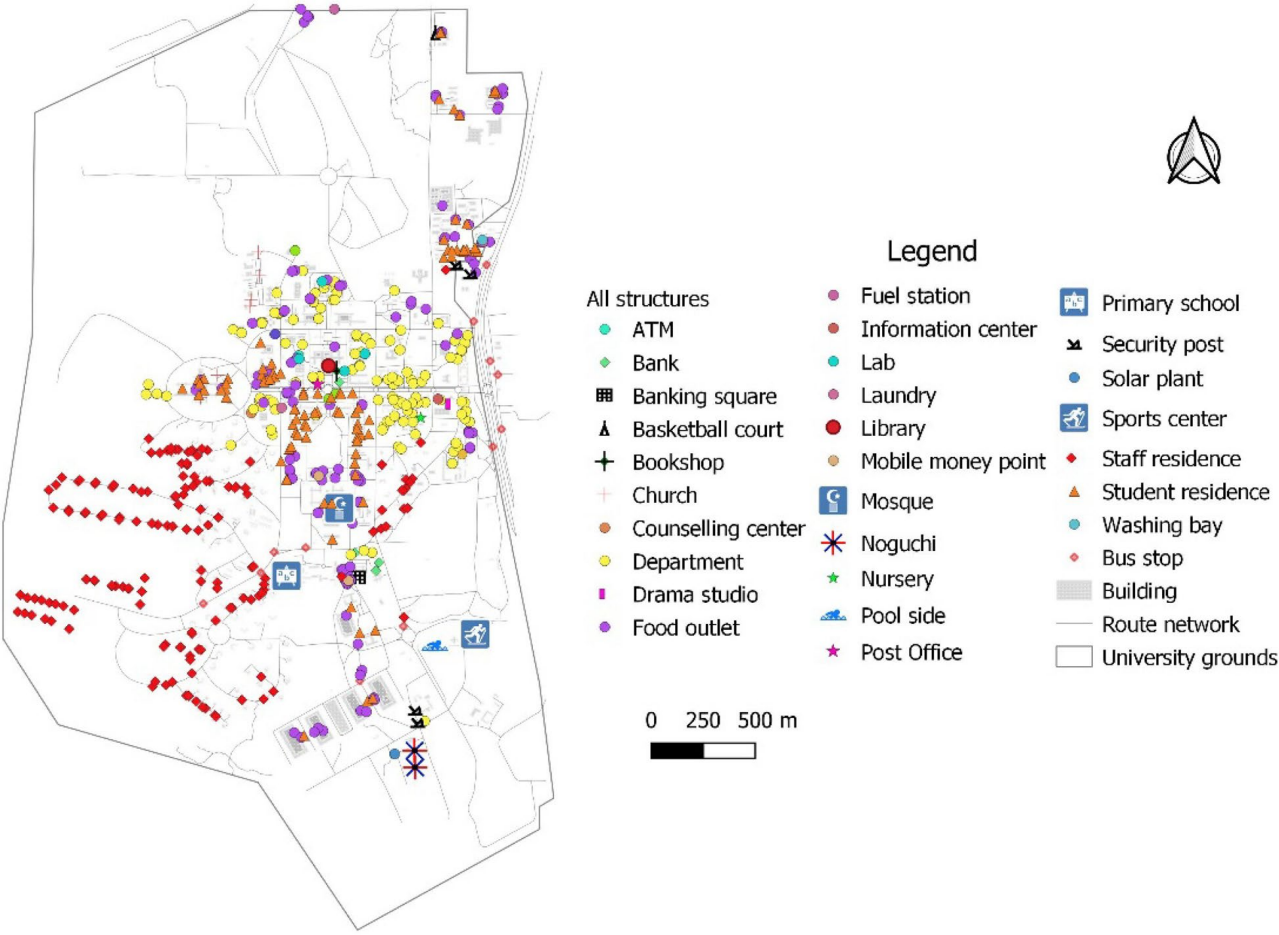
Distribution of structures on the University of Ghana campus

After in-person block-by-block mapping of structures within the within the University boundary, five hundred and fifty-eight (558) structures were identified and mapped. The structures comprised food outlets (138, 24.7%), student hostels/halls (96, 17.2%), lecture halls/other departmental buildings (including administrative offices, conference, or meeting rooms) (124, 22.2%), staff accommodation (154, 27.6%), libraries and bookshops based on the focus of this study. Figure 1 shows the distribution of the various structures. Student hotels/halls (hereinafter, student residence) were mostly large storey/muti-storey buildings compared to staff accommodation which were usually small bungalows. The study focused on university-managed student residences. Rented rooms from private landlords were excluded, as these were farther from the University campus.

### Food outlet characteristics

**Fig. 2:**
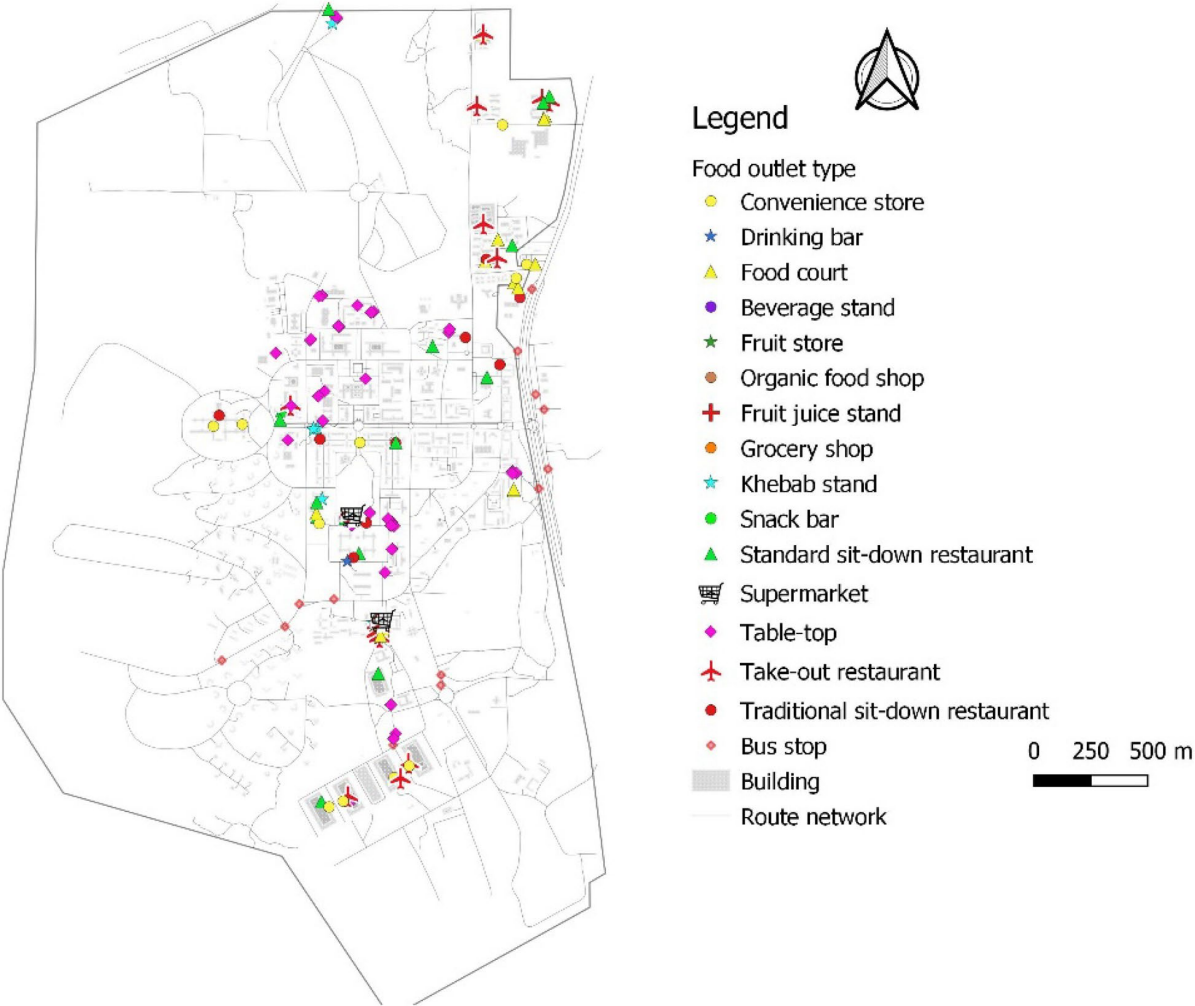
Food outlet typology

Out of 138 food outlets 58% were food service places with 42% being food stores. About 27.5% of all food outlets were table-top operations offering mainly water, carbonated/sugar-sweetened drinks and biscuits; bread with omelets (fried egg); or instant noodles. A few table-top vendors sold fruit, roastgroundnuts and/or water, millet porridge and other cooked breakfast cereals (such as oats). This is followed by traditional/local sit-down restaurants serving prepared meals including mainly rice dishes with vegetable salad (sold separately), *banku/kenkey* with grounded pepper or vegetable sauces (sold separately), *banku/fufu* with soup, and beans with gari/fried plantain (red-red). Together with other food service places (standard sit-down and take-out restaurants), they made up over 35% of food-outlets, suggesting a high prevalence of eating-out among students. Convenience stores had the third highest proportion (11.6%) of food-outlets. In addition to other everyday items, the convenience stores stocked mainly water, soft drinks, biscuits, packaged snacks and other confectionery, and instant noodles. Each hall/hostel of residence had a convenience store inhouse. Interestingly, fruit store (Fruit stores + Fruit juice stand + Organic food shop together) ranked eighth, representing 3.62% of food-outlets. Food-outlets that fell under the Supermarket category were not full-service supermarkets and offered ultra-processed/packaged foods and drinks only, with no fresh food products. Figure 2, Table 3 and Appendix 1 show the typology and the proportion of each food-outlet type in the University foodscape.

**Table 3:** Types of food outlets in

Food outlet type	No	%
Table-top	38	27.54
Traditional sit down	18	13.04
Convenience store	16	11.59
Standard sit-down restaurant	16	11.59
Take-out restaurant/fastfood	15	10.87
Food court	13	9.42
Khebab stand	7	5.07
Grocery shop	5	3.62
Fruit store	3	2.17
Supermarket	2	1.45
Drinking bar	1	0.72
Organic food shop	1	0.72
Fruit juice stand	1	0.72
Snack bar/Ice-cream shop	2	0.72
Total	138	100

**Fig. 3:**
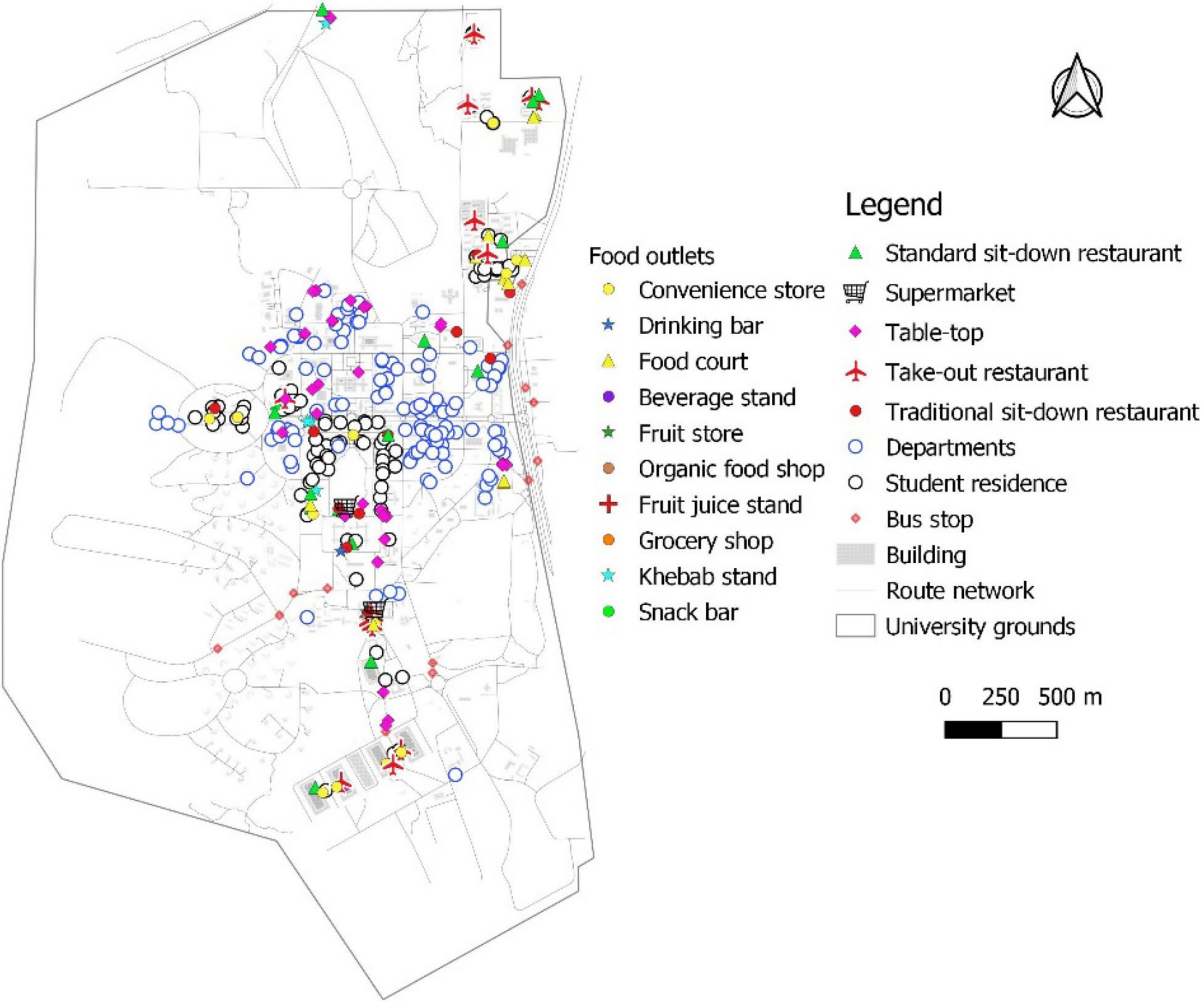
Food outlet types in relation to departmental and residential structures

The distribution of food-outlets was somewhat uneven over the University campus and appeared to be concentrated around the halls/hostels of residence, especially towards the south. There was a limited number of food outlets towards the east where many departmental buildings or lecture halls were located (Fig. 3). These areas were dominated by temporary table-top vendors mainly stocking water, carbonated/SSBs and pastries/biscuits. This may shape the eating habits of students, as the department is where they spend most of their day/time during term-time.

Food delivery service arrangements appeared to offer the opportunity to bridge the distance gap. The study identified four (4) main third-party delivery companies, including “Delivery on Point”, that operated through dispatch riders (on mopeds and motorbikes) stationed at major food-courts who took phone orders from students, procured the food (as requested by student) and delivered it to them. Through dispatch riders, students could buy food from any food outlet or vendor of choice inasmuch as they could afford the cost of delivery. Other food outlets had their own dispatch riders to deliver telephone orders to students. In addition to this, some standard sit-down and take-out/fastfood restaurants like the Basement Plus, Icy cup, Meluv’s Restaurant were listed on online food ordering and delivery platforms like Swyftlyfefood, Bolt food and Jumia food, which also enabled students to order food from outlets both within and outside the University foodscape.

### Healthiness of food outlets

**Fig. 4:**
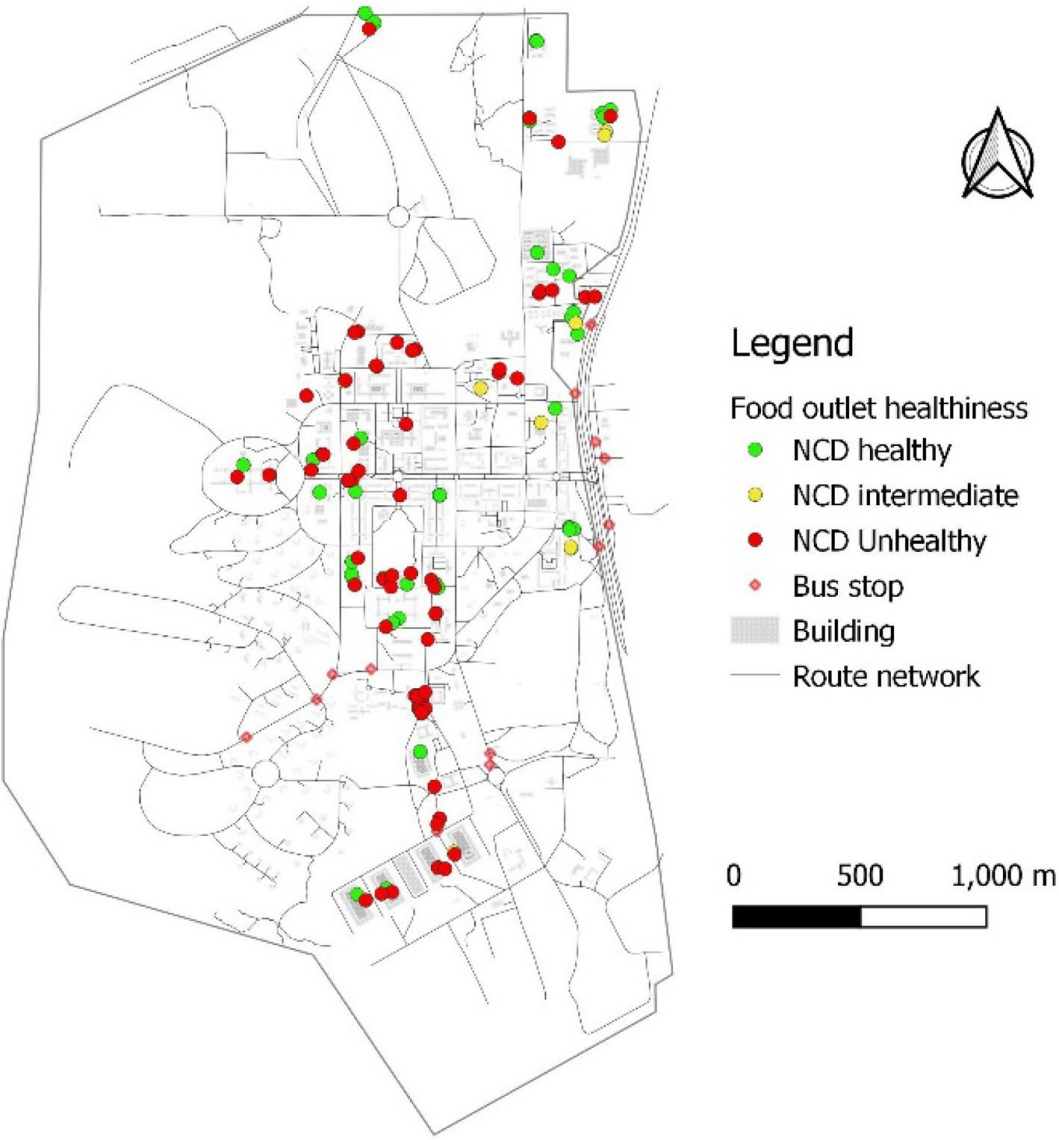
Food outlet healthiness

**Fig. 5:**
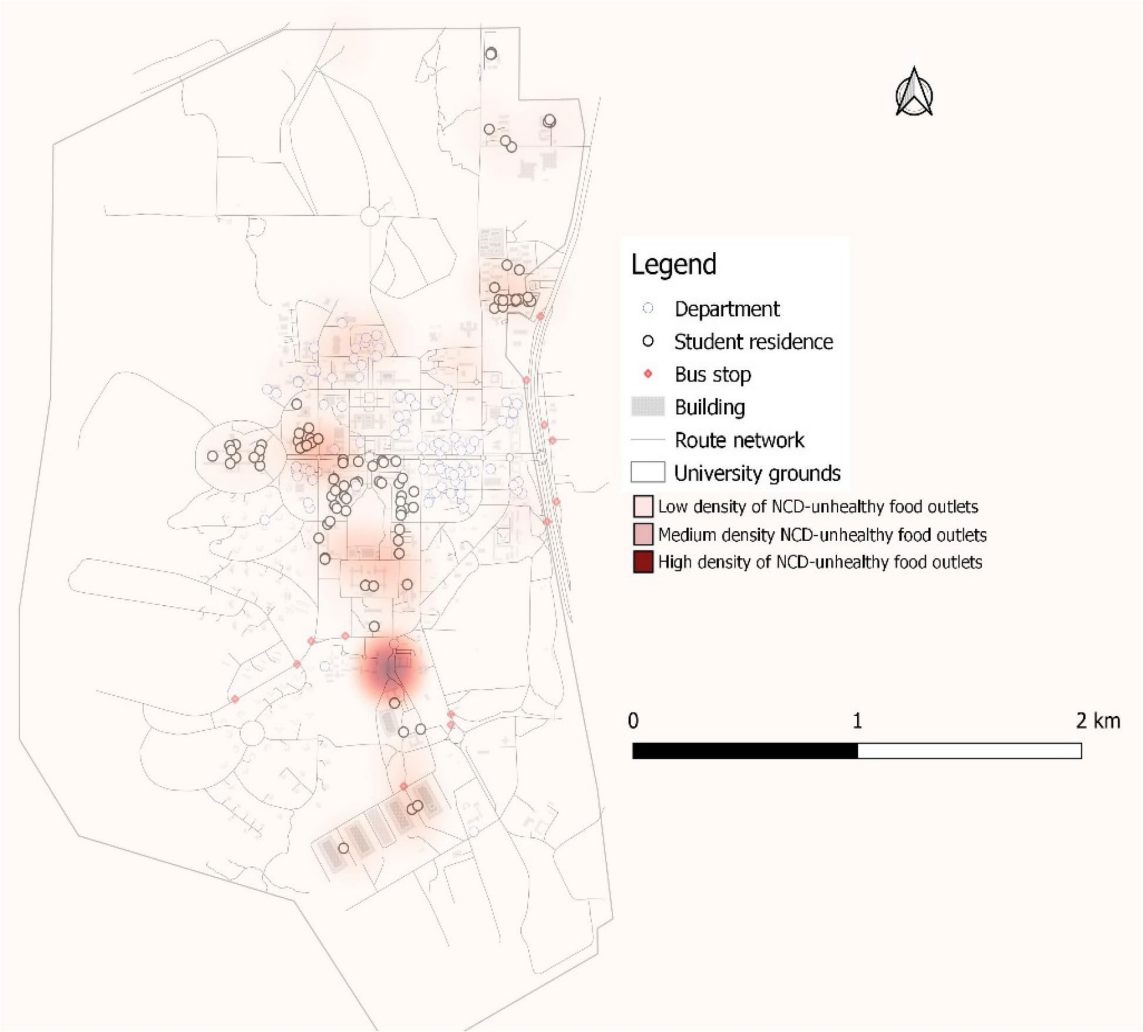
Kernel density map showing concentration of NCD-unhealthy food outlets

**Fig. 6:**
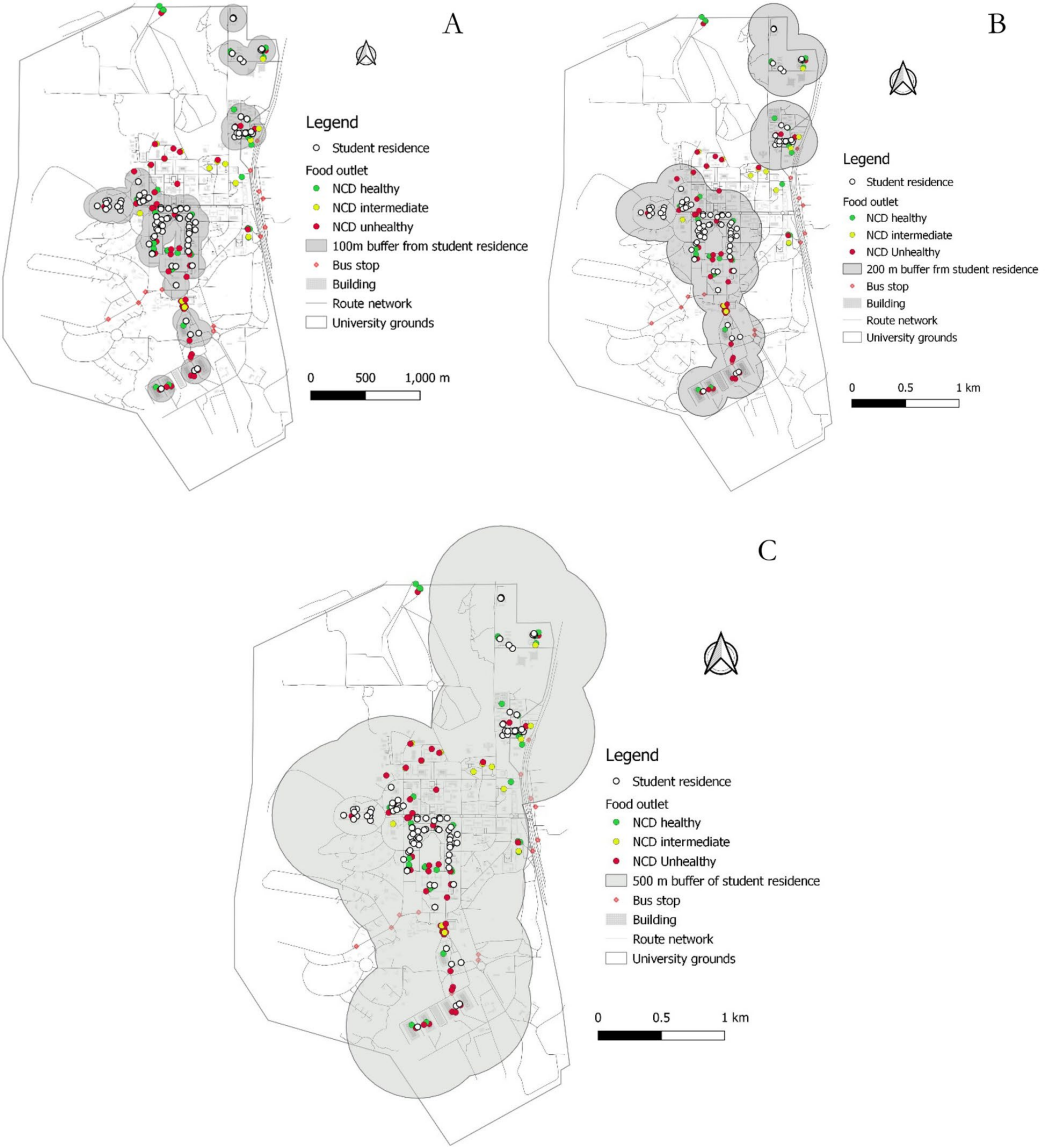
**A**, **B**, **C** Healthiness of food outlets within 100, 200 and 500 m buffer of student residence

Food outlet assessment showed that there were more NCD-unhealthy (50.72%) than NCD-healthy food outlets by nearly 11 percentage points and 9.42% of food outlets being NCD-intermediate. Figure 4 shows the distribution of NCD-healthy and -unhealthy food outlets. The heat map shows the density of NCD-unhealthy food outlets (Fig. 5). The density of NCD-unhealthy food outlets is highest towards the south of the campus followed by parts of the middle belt of the foodscape close to a high number of student residences. About 89%, 89% and 98% of NCD-unhealthy food outlets were respectively within 100 m, 200 m and 500 m buffer of halls/hostels of residence. This compares to about 85%, 85% and 94% of NCD-healthy food outlets within 100, 200, and 500 m of student residence, respectively. See Figs. 6A, B, and C. Statistical analysis showed that the difference between the proportion of NCD-unhealthy food outlets and NCD-healthy food outlets within 100 m buffer of student residencies was statistically significant (*p* < 0.001) but not for food outlets within 200- and 500-m buffer as shown in Table 4.

**Table 4:** Two sample t-tests of NCD-health and NCD-unhealthy food outlets within 100/200/500 m buffer of student halls/hostels

	100-m buffer		200-m buffer		500-m buffer	
	NCD-hfo	NCD-ufo	NCD-hfo	NCD-ufo	NCD-hfo	NCD-ufo
Mean	46.61	61.04	114.39	121.08	282.47	286.52
Variance	874.97	676.39	3342.80	2496.76	17,219.15	15,915.13
Observations	111	120	311	368	1050	1316
df	220		617		2209	
t Stat	-3.92		-1.60		-0.76	
P(T < = t) two-tail	0.001		0.112		0.453	

**Fig. 7:**
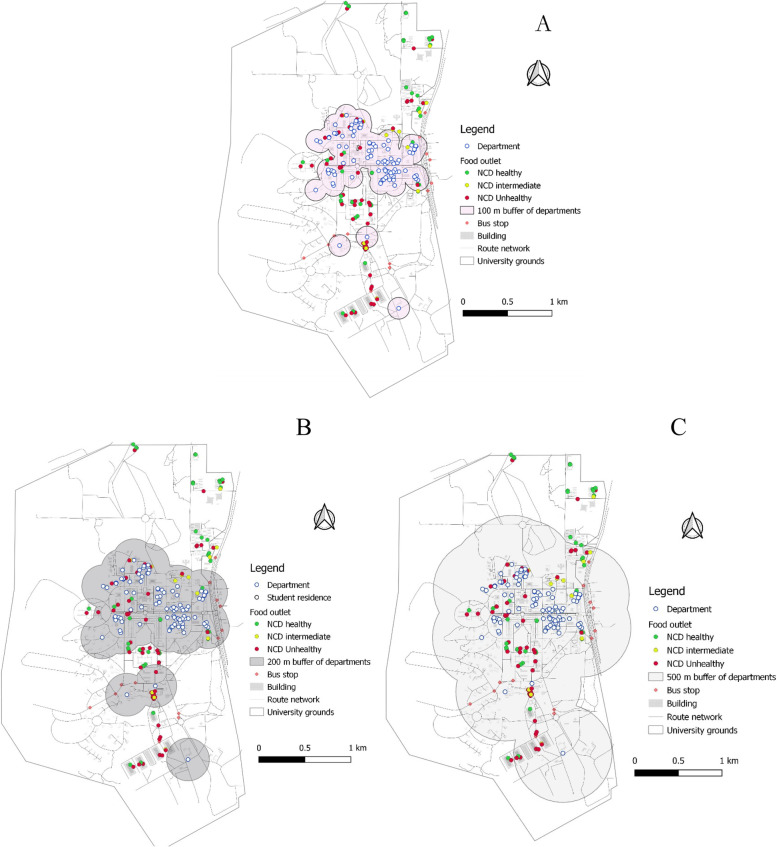
**A**, **B**, **C** Food outlets within 100, 200, 500 m buffer of departmental buildings

Regarding distance between departments and food outlets, 46%, 64% and 94% of NCD-unhealthy food outlets were respectively within 100, 200, and 500 m buffer of departmental buildings/lecture halls (Figs. 7A, B, C), compared to 32%, 48%, and 74% of NCD-healthy food outlets. As shown in Table 5, the differences between NCD-healthy and NCD-unhealthy outlets within 100 m and 500 m buffer of departments were statistically supported at 5% level of significance. These clustering were confirmed in the nearest-neighbour analysis (NNA). The NNA suggested that the average distance between one and another NCD-unhealthy food outlet was approximately 25 m, while the average distance between two NCD-healthy food outlets was 28 m. It is also important to add that more than 42% of all table-top vendors clustered around departmental buildings.

**Table 5:** Two sample t-tests of NCD-health and NCD-unhealthy food outlets within 100, 200, 500 m buffer of departmental buildings

	100-m buffer		200-m buffer		500-m buffer	
	NCD-hfo	NCD-ufo	NCD-hfo	NCD-ufo	NCD-hfo	NCD-ufo
Mean	69.32	63.61	124.44	119.20	311.39	299.61
Variance	581.92	563.21	2161.75	2375.67	12701.69	18559.42
Observations	132	142	383	277	1999	1089
df	272		658		3086	
t Stat	1.98		1.40		2.57	
P(T < = t) two-tail	0.049		0.162		0.010	

## Discussion

This study used Geographic Information System (GIS) to characterise the foodscape in the University of Ghana campus. This is the only study to use GIS methods to characterise the features of the food environment within a Ghanaian university community context. The study identified a total of 138 food-outlets in the University foodscape, which were unevenly distributed. Table-top operations ranked the single-largest food-outlet type (27.53%), although all restaurant types (traditional sit-down (18%), standard sit-down (16%) and take-out restaurants (15%)) put together constituted nearly half of all food-outlets identified. A little over 50% of food-outlets were NCD-unhealthy, about 10 percentage points more common than NCD-healthy food-outlet availability over the foodscape. More food-outlets were clustered around student residences with limited availability of food-outlets around departmental buildings and lecture halls. The kernel-density analysis confirmed that NCD-unhealthy food-outlets also clustered closer to student residences, creating/suggesting an unhealthy food environment. These results suggest that the University foodscape appears to be less healthy by making more unhealthy food options easily accessible (closer) to students.

Food service places dominated the campus foodscape, making up about two-fifths of all outlets identified. This suggests a high eating-out among students in this campus. That is, the prevailing characteristics of the University foodscape may be a materialised reflection of students’ longstanding preferences. Qualitative research among UK and Bangladeshi students have suggested that academic demands on time (among students in this university [34]) and the lack of cooking skills among young university students make eating-out a convenient choice [58, 59]. While all these reasons may not be the case among students at our study university due to differing cultural contexts, the findings here are consistent with the results from an urban poor non-student community in Ghana using the GIS method [5] and the Xi Hu district of China [46] showing a high number of out-of-home cooked food and fast-food restaurants, respectively. Using the Ordnance Survey’s Points of Interest (POI) geo-data and the UK Household Longitudinal Study (UKHLS) Penny et al. (2018) found that a higher density of away-from-home food-outlets around the home/neighbourhood in relation to all other food-outlet types, showed greater odds of UK households expending a higher proportion of their monthly food expenditure eating out [60]. There is convincing evidence associating eating-out (food away from home) with poor diet quality [61–63] and high total energy intake, low micro-nutrient and FV intake (Lachat et al., 2012 [64]; Llanaj et al., 2018 [65]), and high BMI among adolescents and adults [63, 66, 67] including university students [65]. However, in the Dake et al. [5] study conducted in the same city as in the current study, every additional away-from-home food-outlet in the study area was associated with a 0.1 kg/m^2^ less BMI among a non-university adult population. It could be postulated from systematic review evidence [68] that the determinants of away-from-home food consumption in this student community should be considered in future research to inform tailored interventions aimed at reducing the influence of the local foodscape on the adoption of unhealthy dietary behaviours.

The study identified that food-outlets were not evenly spread over the university foodscape. This is consistent with qualitative findings from students in our study university [34] and a non-university context in China in a study that used the GIS method [46]. The study assessed the proximity/clustering of food-outlets around departmental buildings (including lecture rooms) and halls/hostels of residence, as these are the places students spend most of their time on campus. There was limited availability of food-outlets closer to departments, with most food-outlets located around student residencies. Many of the food-outlets around departments were informal temporary table-top structures selling snacks, biscuits other confectionery and carbonated/SSB, similar to the level of prevalence observed in informal communities in South Africa [69] where informal food vendors were the most the popular. Specific to university settings, Pulz et al. [24] used a cross-sectional descriptive design and also found more unhealthy food options and snacks sold at a university’s food service facilities in Brazil. Franco et al. [70] and Barbosa et al. [71] also found that UPFs dominated the campus food environments at the expense of fresh and healthy food options in their assessment of the food environments in different public universities in Brazil. In HICs, similar findings have been reported in historically black USA universities in studies using GIS methods [72]. In cross-sectional surveys [23, 73], direct observations [74], qualitative interviews (Dhillon et al., 2019a [19]; Hilger-Kolb & Diehl, 2019 [21]) have also found the relative availability of less-healthy food options at university campus food venues in Spain, New Zealand, Australia, and the USA, respectively, including vending machines [74, 75].

Although the observed prevalence of informal food-outlets may not be expected in a highly regulated and ‘elite’ community like the study area, there may be cultural and normative underpinnings to this finding. It may encourage frequent ultra-processed food (UPF) consumption and lead to unhealthy dietary habit formation over time [3]—including meal skipping, excess calorie intake. In that, the majority of students spent approximately 50% of their weekday at their departments and returned to their residences later in the afternoon or evening time. Previous research suggest that food-outlets consistently encountered by individuals in their daily routine and commuting routes trigger intentions to eat/buy and shape choice and preferences, including emotional attachment to particular food-outlets (Burgoine et al., 2013 [76]; Clary et al., 2017 [3]). However, a systematic review found limited evidence associating characteristics of the school food environment with students’ food consumption patterns [77]. Indeed, there was no included study from an African setting. It is suggested that universities in the study country take advantage of their powers as semi-autonomous bodies to consider healthy school foodscape interventions (especially at departments) to lead the way in national efforts towards creating healthy food environments around the home, work, and other school environments.

Another important finding from our analysis showed a higher proportion (over 50%) of NCD-unhealthy food outlets than NCD-healthy food-outlets available within the University foodscape. These outlets including traditional sit-down and take-out restaurants, convenience stores and table-top vendors offered energy-dense meals, deep-fried (fatty) foods, SSBs and other UPFs, and operated for long hours. A similar pattern of distribution between healthy and unhealthy food-outlets have been reported in both high- and low-income countries (Bodor et al., 2010 [78]; Maimaiti et al., 2020 [46]; Patel et al., 2017 [79]; Zhang & Huang, 2018 [80]), including Ghana [5]. In this study, many of such NCD-unhealthy food-outlets clustered closer to student residencies and dominated the food market around the residencies (as evident on the heat map in Fig. 5 and buffer maps from departmental buildings (Figs. 7A, B& C)) in the face of limited availability of grocery shops stocking organic/inorganic FV. None of the two identified supermarkets were full-service, stocking mainly ultra-processed/packaged foods only with no fresh food products. The availability of unhealthy food-outlets (such as SSB vending machines, fast-food, convenience stores) within 1 km buffer of schools has been found to increase the consumption of SSB’s [81], deep-fried salty snacks, junk foods or other unhealthy foods and low FV intake among students in Canada [81], the UK [82], Ireland [83], and Brazil [84]. Neighbourhoods with relatively limited outlets stocking fresh food options have been associated with significantly low FV consumption [85, 86] and healthy food consumption in general [87]. In Ghana, for every other convenience store in a poor non-student urban community in the same city as this study, BMI in persons aged 15–59 years increased by 0.2 kg/m^2^ [5]. In this study, the long operating hours of outlets (including convenience shops, table-top vendors selling bread with omelets and kelewele, and khebab) closer to student residences may encourage less healthy late-night eating habits among students. Further research could consider the influence of this campus’ food environment features on students’ food choice and eating behaviours.

Most studies characterising the food environment have focused on a limited selection of food-outlet types. This study included all food-outlet types available within the study area. Another strength of this study is the use of a robust classification instrument to categorise all available food-outlets as NCD-healthy, NCD-intermediate or NCD-unhealthy based on relevant research [50, 55, 56, 87].

Despite the strengths, this study had limitations. There was bad satellite imagery in sections of the study area during the online mapping and validation to create the study’s basemap. A few of the tasks on the University of Ghana campus HOT Tasking Manager appeared invisible because there was cloud cover obstruction. Those parts could not be digitised and some structures may have been missed at the online mapping stage. However, such structures would have been captured during structure verification/ground-truthing, highlighting the rationale for employing a systematic approach involving the combination of block-by-block observation, respondent reports, and a GPS in this study to capture the characteristics of the food environment.

A second limitation could be the geographical inaccessibility encountered during the ground-truthing exercise. Access into some residential areas like the Vice Chancellor’s area and the University Guest Houses, were not allowed for security reasons. As this could mean some outlets missing from the maps, its impact on the study’s findings would be negligible, given that students were not allowed access into these areas.

In close relation to the above, the study may not have captured all outlets within the university foodscape given that some food vendors were mobile. This includes vendors who ported FV, pastries, ice creams, and snacks on their heads; or carted ice cream, SSBs, and pastries on bicycles to various points of the foodscape. However, the mobile food vendors constituted a small proportion of food resources in the study community to make a significant difference to the findings reported here.

Another limitation could be issues encountered during the upload of fieldpaper sheets to Fieldpaper.org after ground-truthing. Images of some updated fieldpaper sheets captured using the Samsung Galaxy S5 and Alcatel-3 V phone cameras could not be uploaded to Fieldpapers.org. Those parts of the map were therefore manually updated online with information captured on the fieldpapers during structure verification using the individual fieldpaper codes. This may have affected the accuracy of the point location for the structures entered manually. Although this was the case for an insignificant number of the structures identified and mapped, the findings of the study should be interpreted in the light of this and the foregoing limitations.

Many food items become unhealthy when consumed beyond recommended levels. Thus some of the food items used as markers of healthiness may become risks to personal health if not consumed in the most appropriate and recommended manner [88]. Therefore, the food outlet healthiness categorisations—NCD-healthy, NCD-intermediate, NCD-unhealthy—developed in this study should be interpreted with circumspection and in the proper context.

## Conclusion

This study set out to identify the features of a university food environment and to assess the healthiness of the food-outlets within the campus foodscape. The campus foodscape offers a variety of food resources but an imbalance in the distribution of the food-outlets was identified. However, there was a robust food delivery system with some food service places listed on online food ordering and delivery platforms. Nearly half of the food resources qualified as NCD-unhealthy food outlets which clustered around student halls/hostels of residence, suggesting a less healthy food university environment. Overall, our findings have important implications for further research and policy interventions towards identifying environmental risk factors associated with unhealthy food behaviours, overweight/obesity, NCDs, and other health outcomes among university students in Ghana during term-time. Another distinctive contribution of this study is the provision of evidence on how remotely mapped data in a collaborative mapping approach can be combined with direct observation and other survey methods to produce data that provides useful insights into relationships between people and location characteristics. This would provide methodological guidance to other investigators, particularly, under-resourced researchers.

## Supplementary Information

**Additional file 1. Appendix 1.**Typology of food-outlets identified in the University foodscape.

## Data Availability

The datasets used in this study, except identifiable personal information, are available from the corresponding author on reasonable request.

## Footnotes

1 Commonwealth Hall (the only male hall of residence); Akuafo Hall; Mensah Sarbah Hall; Volta Hall (the only female hall of residence); Jubilee Hall; and Legon Hall.

2 Alexander Kwapong Hall, Hilla Limann Hall, Jean Nelson Hall, Africa Union Hall, Bani Hall, Elizabeth Sey Hall, Evandy Hall, James Topp Nelson Yankah Hall (source: https://africavarsities.com/university-of-ghana/).
