# Supplementary material for: Type, density, and healthiness of food-outlets in a university foodscape: a geographical mapping and characterisation of food resources in a Ghanaian university campus
Source: BMC Public Health. 2022 Oct 13;22:1912. doi: 10.1186/s12889-022-14266-7 (PMC9563792; doi:10.1186/s12889-022-14266-7)
Supplement: Supplementary file 1 — Additional file 1. Appendix 1.Typology of food-outlets identified in the University foodscape. [file 12889_2022_14266_MOESM1_ESM.docx]

# Appendices

Appendix 1: Typology of food-outlets identified in the University foodscape

| **Food outlet type** | **Food store or service place** | **Description** |
| --- | --- | --- |
| Convenience store | Food store | Formal structure stocking packaged or ready-to-eat food options together with everyday/ general consumer goods. Usually small-sized and stock limited variety of items. Located closer/within residence of patrons. Open extended hours. One or two staff working at a time. (Maimaiati et al., 2019) |
| Grocery shop | Food store | Wooden/formal structure that specialised in stocking fresh and packaged food products, and non-food products. Usually stocked a relatively bigger variety. |
| Fruit store | Food store | Wooden/formal structure that specialises in stocking fresh fruits only. |
| Supermarket | Food store | Formal structure, specialised in stocking packaged foods, and non-food products. Relatively bigger than a grocery shop and stocks a wider variety. Usually has more than one point-of-sale device/point and a bigger staff size than convenience store. |
| Organic food shop | Food store | Usually grocery-shop style that specialised in selling only/mainly organic food products. |
| Traditional sit down | Food service place | Usually operates in wooden structure or open space. Shared wooden or metal benches and tables are usually used, creating an informal (atmosphere) and a local/old-style set up—commonly called the 'Chop-bar' style. Plastic tables and chairs are becoming more common. This encourages eating at the premises. Food was served in plastic and/or earthenware bowls. Usually served soupy dishes like fufu, banku, kokonte, TZ and occasionally some rice or yam dishes. |
| Standard sit-down restaurant | Food service place | Operated in formal structures with formal/ semi-formal set-up and seating with chefs and smartly dressed waiters. Table number system, receipt/invoicing system were used. Served a combination of select local and continental dishes using ceramic plates, cutlery sets. Offered take-away packing option at customers’ request. |
| Take-out restaurant/fastfood | Food service place | Formal/semi-formal enclosed structure and/or counter, specialises in food prepared to be taken away for consumption elsewhere than at the premises. |
| khebab stand | Food service place | Kiosks, other wooden/metallic structure, stand-alone barbeque grill specialising in preparing khebab or other grilled meat. Usually operated extended hours. |
| Drinking bar | Food service place | Formal/semi-formal permanent enclosed structure that specialised in selling alcoholic and/or non-alcoholic (carbonated/sweetened) drinks with seating for drinking at the premises. Usually operated extended hours. |
| Fruit juice stand | Food service place | Temporary or permanent structure that prepared 100% natural fruit/vegetable juices or smoothies. |
| Snack bar/Ice-cream shop | Food service place | Permanent structure that specialised in selling ice creams and/or other (mostly frozen) snacks only. |
| Table-top | Both | Outlets operating from tables or table-like non-permanent structures mostly selling salted/sweetened snacks, carbonated/sugar-sweetened beverages/other confectionery, and bread with egg (omelette). |
| Food court | Both | Wet-market style food outlets having three or more different vendors with each offering different types of foods. |
